# Readiness assessments for gender-affirming surgical treatments: A systematic scoping review of historical practices and changing ethical considerations

**DOI:** 10.3389/fpsyt.2022.1006024

**Published:** 2022-10-20

**Authors:** Travis Amengual, Kaitlyn Kunstman, R. Brett Lloyd, Aron Janssen, Annie B. Wescott

**Affiliations:** ^1^Department of Psychiatry and Behavioral Sciences, Northwestern Medicine, Chicago, IL, United States; ^2^The Pritzker Department of Psychiatry and Behavioral Health, Ann & Robert H. Lurie Children’s Hospital of Chicago, Chicago, IL, United States; ^3^Galter Health Science Library, Northwestern University, Chicago, IL, United States

**Keywords:** gender-affirming surgery, standards of care, world professional association for transgender health, ethics, informed consent, transgender and gender diverse (TGD), mental health, scoping review

## Abstract

Transgender and gender diverse (TGD) are terms that refer to individuals whose gender identity differs from sex assigned at birth. TGD individuals may choose any variety of modifications to their gender expression including, but not limited to changing their name, clothing, or hairstyle, starting hormones, or undergoing surgery. Starting in the 1950s, surgeons and endocrinologists began treating what was then known as transsexualism with cross sex hormones and a variety of surgical procedures collectively known as sex reassignment surgery (SRS). Soon after, Harry Benjamin began work to develop standards of care that could be applied to these patients with some uniformity. These guidelines, published by the World Professional Association for Transgender Health (WPATH), are in their 8th iteration. Through each iteration there has been a requirement that patients requesting gender-affirming hormones (GAH) or gender-affirming surgery (GAS) undergo one or more detailed evaluations by a mental health provider through which they must obtain a “letter of readiness,” placing mental health providers in the role of gatekeeper. WPATH specifies eligibility criteria for gender-affirming treatments and general guidelines for the content of letters, but does not include specific details about what must be included, leading to a lack of uniformity in how mental health providers approach performing evaluations and writing letters. This manuscript aims to review practices related to evaluations and letters of readiness for GAS in adults over time as the standards of care have evolved *via* a scoping review of the literature. We will place a particular emphasis on changing ethical considerations over time and the evolution of the model of care from gatekeeping to informed consent. To this end, we did an extensive review of the literature. We identified a trend across successive iterations of the guidelines in both reducing stigma against TGD individuals and shift in ethical considerations from “do no harm” to the core principle of patient autonomy. This has helped reduce barriers to care and connect more people who desire it to gender affirming care (GAC), but in these authors’ opinions does not go far enough in reducing barriers.

## Introduction

Transgender and gender diverse (TGD) are terms that refer to any individual whose gender identity is different from their sex assigned at birth. Gender identity can be expressed through any combination of name, pronouns, hairstyle, clothing, and social role. Some TGD individuals wish to transition medically by taking gender-affirming hormones (GAH) and/or pursuing gender-affirming surgery (GAS) (1).^1^ The medical community’s comfort level with TGD individuals and, consequently, their willingness to provide a broad range of gender affirming care (GAC)^2^ has changed significantly over time alongside an increasing understanding of what it means to be TGD and increasing cultural acceptance of LGBTQI people.

Historically physicians have placed significant barriers in the way of TGD people accessing the care that we now know to be lifesaving. Even today, patients wishing to receive GAC must navigate a system that sometimes requires multiple mental health evaluations for procedures, that is not required of cisgender individuals.

The medical and psychiatric communities have used a variety of terms over time to refer to TGD individuals. The first and second editions of DSM described TGD individuals using terms such as transvestism (TV) and transsexualism (TS), and often conflated gender identity with sexuality, by including them alongside diagnoses such as homosexuality and paraphilias. Both the DSM and the International Classification of Diseases (ICD) have continuously changed diagnostic terminology and criteria involving TGD individuals over time, from Gender Identity Disorder in DSM-IV to Gender Dysphoria in DSM-5 to Gender Incongruence in ICD-11.

In 1979, the Harry Benjamin International Gender Dysphoria Association^3^, renamed the World Profession Association for Transgender Health (WPATH) in 2006, was the first to publish international guidelines for providing GAC to TGD individuals. The WPATH Standards of Care (SOC) are used by many insurance companies and surgeons to determine an individual’s eligibility for GAC. Throughout each iteration, mental health providers are placed in the role of gatekeeper and tasked with conducting mental health evaluations and providing required letters of readiness for TGD individuals who request GAC (1). As part of this review, we will summarize the available literature examining the practical and ethical changes in conducting mental health readiness assessments and writing the associated letters.

While the WPATH guidelines specify eligibility criteria for GAC and a general guide for what information to include in a letter of readiness, there are no widely agreed upon standardized letter templates or semi-structured interviews, leading to a variety of practices in evaluation and letter writing for GAC (2). To our knowledge, this is the first scoping review to summarize the available research to date regarding the evolution of the mental health evaluation and process of writing letters of readiness for GAS. By summarizing trends in these evaluations over time, we aim to identify best practices and help further guide mental health professionals working in this field.

## Methods

**FIGURE 1:**
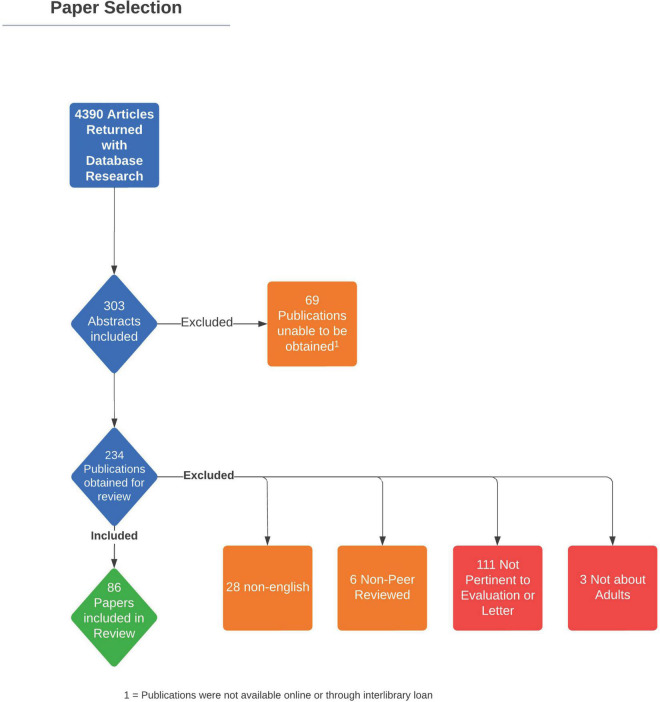
PRISMA flow diagram demonstrating article review process.

The review authors conducted a comprehensive search of the literature in collaboration with a research librarian (ABW) according to PRISMA guidelines. The search was comprised of database-specific controlled vocabulary and keyword terms for (1) mental health and (2) TGD-related surgeries. Searches were conducted on December 2, 2020 in MEDLINE (PubMed), the Cochrane Library Databases (Wiley), PsychINFO (EBSCOhost), CINAHL (EBSCOhost), Scopus (Elsevier), and Dissertations and Theses Global (ProQuest). All databases were searched from inception to present without the use of limits or filters. In total, 8,197 results underwent multi-pass deduplication in a citation management system (EndNote), and 4,411 unique entries were uploaded to an online screening software (Rayyan) for title/abstract screening by two independent reviewers. In total, 303 articles were included for full text screening (Figure 1), however, 69 of those articles were excluded as they were unable to be obtained online or through interlibrary loan. Both review authors conducted a full text screen of the remaining 234 articles. Articles were included in the final review if they specified criteria used for mental health screening/evaluation and/or letter writing for GAS, focused on TGD adults, were written in English, and were peer-reviewed publications. Any discrepancies were discussed between the two review authors TA and KK and a consensus was reached. A total of 86 articles met full inclusion criteria. Full documentation of all searches can be found in the Supplementary material.

## Results

In total, 86 articles were included for review. Eleven articles were focused on ethical considerations while the remaining 75 articles focused on the mental health evaluation and process of writing letters of readiness for GAS. Version 8 of the SOC was published in September of 2022 during the review process of this manuscript and is also included as a reference and point of discussion.

### Prior to the publication of the standards of care

Fourteen articles were identified in the literature search as published prior to the development of the WPATH SOC version 1 in 1979. Prominent themes included classification, categorization, and diagnosis of TS. Few publications described the components of a mental health evaluation, and inclusion and exclusion criteria, for GAS. Many publications focused exclusively on transgender females, with a paucity of literature examining the experiences of transgender males during this timeframe.

Authors emphasized accurate diagnosis of TS, highlighting elements of the psychosocial history including early life cross-dressing, preference for play with the opposite gender toys and friends, and social estrangement around puberty (3). One author proposed the term gender dysphoria syndrome, which included the following criteria: a sense of inappropriateness in one’s anatomically congruent sex role, that role reversal would lead to improvement in discomfort, homoerotic interest and heterosexual inhibition, an active desire for surgical intervention, and the patient taking on an active role in exploring their interest in sex reassignment (4). Many authors attempted to differentiate between the “true transsexual” and other diagnoses, including idiopathic TS; idiopathic, essential, or obligatory homosexuality; neuroticism; TV; schizophrenia; and intersex individuals (5, 6).

Money argued that the selection criteria for patients requesting GAS include a psychiatric evaluation to obtain collateral information to confirm the accuracy of the interview, work with the family to foster support of the individual, and proper management of any psychiatric comorbidities (5). Authors began to assemble a list of possible exclusion criteria for receiving GAS such as psychosis, unstable mental health, ambivalence, and secondary gain (e.g., getting out of the military), lack of triggering major life events or crises, lack of sufficient distress in therapy, presence of marital bonds (given the illegality of same-sex marriage during this period), and if natal genitals were used for pleasure (3–5, 7–13).

Others focused the role of the psychiatric evaluation on the social lives and roles of the patient. They believed the evaluation should include exploring the patient’s motivation for change for at least 6–12 months (8), facilitating realistic expectations of treatment, managing family issues, providing support during social transition and post-operatively (13), and encouraging GAH and the “real-life test” (RLT). The RLT is a period in which a person must fully live in their affirmed gender identity, “testing” if it is right for them. In 1970, Green recommended that a primary goal of treatment was that, “the male patient must be able to pass in society as a socially acceptable woman in appearance and to conduct the normal affairs of the day without arousing undue suspicion” (14). Benjamin also noted concern that “too masculine” features may be a contraindication to surgery so as to not make an “acceptable woman” (7). Some publications recommended at least 1–2 years of a RLT (3, 7, 11, 15), while others recommended at least 5 years of RLT prior to considering GAS (12). Emphasis was placed on verifying the accuracy of reported information from family or friends to ensure “authentic” motivation for GAS and rule out ambivalence or secondary gain (e.g., getting out of the military) (10).

Ell recommended evaluation to ensure the patient has “adequate intelligence” to understand realistic expectations of surgery and attempted to highlight the patient’s autonomy in the decision to undergo GAS. He wrote, “That is your decision [to undergo surgery]. It’s up to you to prove that you are a suitable candidate for surgery. It’s not for me to offer it to you. If you decide to go ahead with your plans to pass in the opposite gender role, you do it on your own responsibility” (8). Notably, many authors conceptualized gender transition along a binary, with individuals transitioning from one end to the other.

In these earliest publications, one can start to see the beginning framework of modern-day requirements for accessing GAS, including ensuring an accurate diagnosis of gender incongruence; ruling out other possible causes of presentation such as psychosis; ensuring general mental stability; making sure that the patient has undergone at least some time of living in their affirmed gender; and that they are able to understand the consequences of the procedure.

### Standards of care version 1 and 2

#### Changes to the standards of care

**FIGURE 2:**
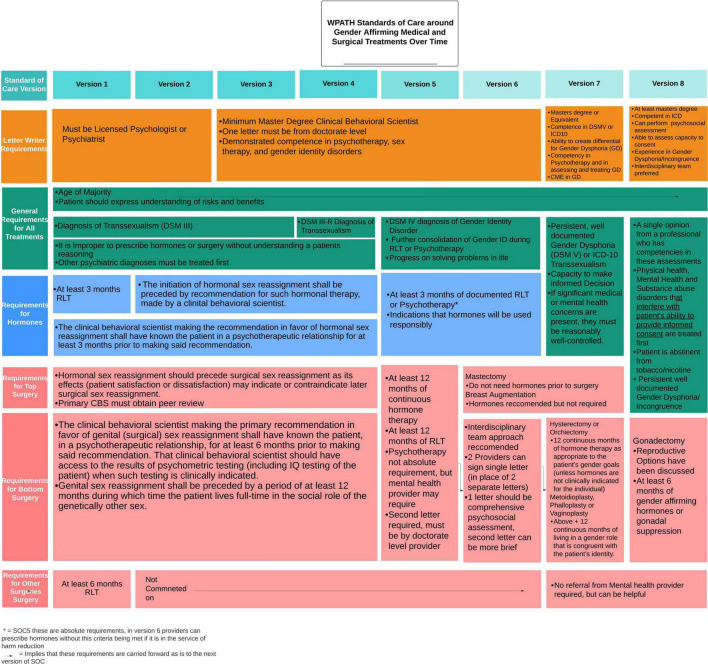
Changes to the World Professional Association for Transgender Health (WPATH) standards of care around gender affirming medical and surgical treatments over time.

The first two versions of the WPATH SOC were written in 1979 and 1980, respectively and are substantially similar to one another. SOC version three was the first to be published in an academic journal in 1985 and changes from the first two versions were documented within this publication. The first two versions required that all recommendations for GAC be completed by licensed psychologists or psychiatrists. The first version recommended that patients requesting GAH and non-genital GAS, spend 3 and 6 months, respectively, living full time in their affirmed gender. These recommendations were rescinded in subsequent versions (16). Figure 2 reviews changes to the recommendations for GAC within the WPATH SOC over time.

#### Results review

Five articles published between 1979 and 1980 were included in this review. Again, emphasis was placed on proper diagnosis, classification and consistency of gender identity over time (17, 18).

Wise and Meyer explored the concept of a continuum between TV and TS, describing that those who experienced gender dysphoria often requested GAS, displayed evidence of strong cross-dressing desires with arousal, history of cross-gender roles, and absence of manic-depressive or psychotic illnesses (19). Requirements for GAS at the Johns Hopkins Gender Clinic included at least 2 years of cross-dressing, working in the opposite gender role, and undergoing treatment with GAH and psychotherapy (19). Bernstein identified factors correlated with negative GAS outcomes including presence of psychosis, drug abuse, frequent suicide attempts, criminality, unstable relationships, and low intelligence level (18). Lothstein stressed the importance of correct diagnosis, “since life stressors may lead some transvestites to clinically present as transsexuals desiring SRS” (20). Levine reviewed the diagnostic process employed by Case Western Reserve University Gender Identity Clinic which involved initial interview by a social worker to collect psychometric testing, followed by two independent psychiatric interviews to obtain the developmental gender history, understand treatment goals, and evaluate for underlying co-morbid mental health diagnoses, with a final multidisciplinary conference to integrate the various evaluations and develop a treatment plan (21).

### Standards of care version 3

#### Changes to the standards of care

Version 3 broadened the definition of the clinician thereby broadening the scope of providers who could write recommendation letters for GAC. Whereas prior SOC required letters from licensed psychologists or psychiatrists, version 3 allowed initial evaluations from providers with at least a Master’s degree in behavioral science, and when required, a second evaluation from any licensed provider with at least a doctoral degree. Version 3 recommended that all evaluators demonstrate competence in “gender identity matters” and must know the patient, “in a psychotherapeutic relationship,” for at least 6 months (16). Version 3 relied on the definition of TS in DSM-III, which specified the sense of discomfort with one’s anatomic sex be “continuous (not limited to a period of stress) for at least 2 years” and be independently verified by a source other than the patient through collateral or through a longitudinal relationship with the mental health provider (16). Recommendation of GAS specifically required at least 6–12 months of RLT, for non-genital and genital GAS, respectively (16).”

#### Results review

Nine articles were published during the timeframe that the SOC version 3 were active (1981–1990). Themes in these publications included increasing focus on selection criteria for GAS and emphasis on the RLT, which was used to ensure proper diagnosis of gender dysphoria. Recommendations for the duration of the RLT ranged anywhere between 1 and 3 years (22, 23).

Proposed components of the mental health evaluation for GAS included a detailed assessment of the duration, intensity, and stability of the gender dysphoria, identification of underlying psychiatric diagnoses and suicidal ideation, a mental status examination to rule out psychosis, and an assessment of intelligence (e.g., IQ) to comment on the individual’s “capacity and competence” to consent to GAC. The Minnesota Multiphasic Personality Inventory (MMPI), Weschler Adult Intelligence Scale (WAIS), and Lindgren-Pauly Body Image Scale were also used during assessments (24).

Authors developed more specific inclusion and exclusion criteria for undergoing GAS with inclusion criteria including age 21 or older, not legally married, no pending litigation, evidence of gender dysphoria, completion of 1 year of psychotherapy, between 1 and 2 years RLT with ability to “pass convincingly” and “perform successfully” in the opposite gender role, at least 6 months on GAH (if medically tolerable), reasonably stable mental health (including absence of psychosis, depression, alcoholism and intellectual disability), good financial standing with psychotherapy fees (25), and a prediction that GAS would improve personal and social functioning (26–29). A 1987 survey of European psychiatrists identified their most common requirements as completion of a RLT of 1–2 years, psychiatric observation, mental stability, no psychosis, and 1 year of GAH (27).

### Standards of care version 4

#### Changes to the standards of care

World Professional Association for Transgender Health SOC version four was published in 1990. Between version three and version four, DSM-III-R was published in 1987. Version four relied on the DSM-III-R diagnostic criteria for TS as opposed to the DSM-III criteria in version three. The DSM-III-R criteria for TS included a “persistent discomfort and sense of inappropriateness about one’s assigned sex,” “persistent preoccupation for at least 2 years with getting rid of one’s primary and secondary sex characteristics and acquiring the sex characteristics of the other sex,” and that the individual had reached puberty (30). Notable changes from the DSM-III criteria include specifying a time duration for the discomfort (2 years) and designating that individuals must have reached puberty.

#### Results review

Six articles were published between 1990 and 1998 while version four was active. Earlier trends continued including emphasizing proper diagnosis of gender dysphoria (31, 32), however, a new trend emerged toward implementing more comprehensive evaluations, with an emphasis on decision making, a key element of informed consent.

Bockting and Coleman, in a move representative of other publications of this era, advocated for a more comprehensive approach to the mental health evaluation and treatment of gender dysphoria. Their treatment model was comprised of five main components: a mental health assessment consisting of psychological testing and clinical interviews with the individual, couple, and/or family; a physical examination; management of comorbid disorders with pharmacotherapy and/or psychotherapy; facilitation of identity formation and sexual identity management through individual and group therapy; and aftercare consisting of individual, couple, and/or family therapy with the option of a gender identity consolidation support group. Psychoeducation was a main thread throughout the treatment model and a variety of treatment “subtasks” such as understanding decision making, sexual functioning and sexual identity exploration, social support, and family of origin intimacy were identified as important. The authors advocated for “a clear separation of gender identity, social sex role, and sexual orientation which allows a wide spectrum of sexual identities and prevents limiting access to GAS to those who conform to a heterosexist paradigm of mental health” (33).

This process can be compared with the Italian SOC for GAS which recommend a multidisciplinary assessment consisting of a psychosocial evaluation and informed consent discussion around treatment options, procedures, and risks. Requirements included 6 months of psychotherapy prior to initiating GAH, 1 year of a RLT prior to GAS, and provision of a court order approving GAS, which could not be granted any sooner than 2 years after starting the process of gender transition. Follow-up was recommended at 6, 12, and 24 months post-GAS to ensure psychosocial adjustment to the affirmed gender role (34).

Other authors continued to refine inclusion and exclusion criteria for GAS by surveying the actual practices of health centers. Inclusion criteria included those who had life-long cross gender identification with inability to live in their sex assigned at birth; a 1–2 years RLT (a nearly universal requirement in the survey); and ability to pass “effortlessly and convincingly in society”; completed 1 year of GAH; maintained a stable job; were unmarried or divorced; demonstrated good coping skills and social-emotional stability; had a good support system; and were able to maintain a relationship with a psychotherapist. Exclusion criteria included age under 21 years old, recent death of a parent (35), unstable gender identity, unstable psychosocial circumstances, unstable psychiatric illness (such as schizophrenia, suicide attempts, substance abuse, intellectual disability, organic brain disorder, AIDS), incompatible marital status, criminal history/activity or physical/medical disability (36).

The survey indicated some programs were more lenient around considering individuals with bipolar affective disorder, the ability to pass successfully, and issues around family support. Only three clinics used sexual orientation as a factor in decision for GAS, marking a significant change in the literature from prior decades. Overall, the authors found that 74% of the clinics surveyed did not adhere to WPATH SOC, instead adopting more conservative policies (36).

### Standards of care version 5

#### Changes to the standards of care

**FIGURE 3:**
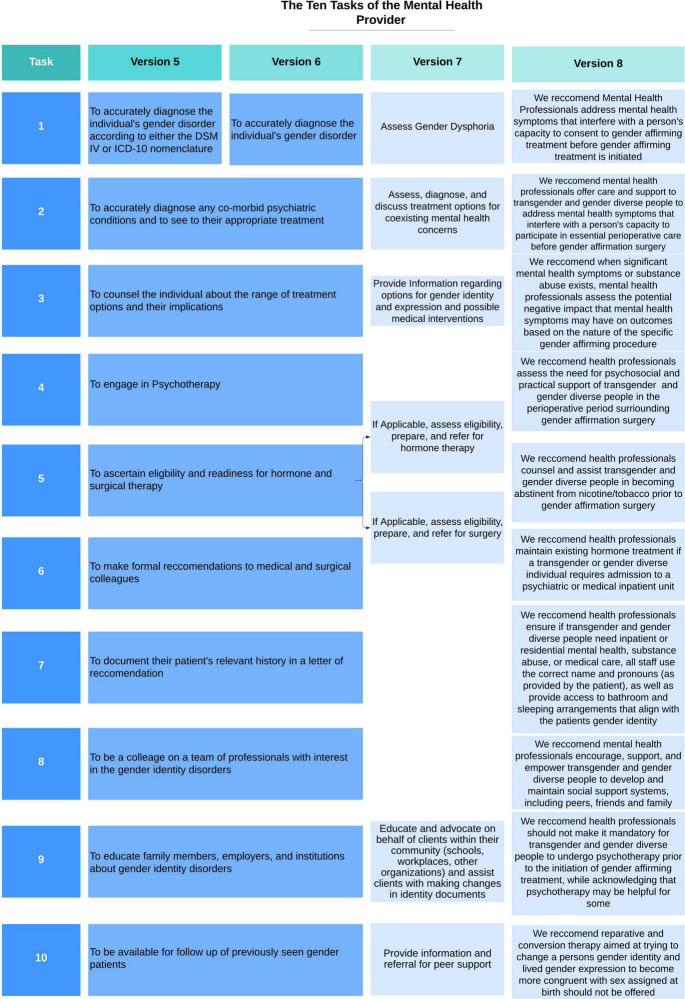
Changes to the ten tasks of the mental health provider within the World Professional Association for Transgender Health (WPATH) standards of care over time.

Published in 1998, version five defined the responsibilities of the mental health professional which included diagnosing the gender disorder, diagnosing and treating co-morbid psychiatric conditions, counseling around GAC, providing psychotherapy, evaluating eligibility and readiness criteria for GAC, and collaborating with medical and surgical colleagues by writing letters of recommendation for GAC (Figure 3). Eligibility and readiness criteria were more explicitly described in this version to refer to the specific objective and subjective criteria, respectively, that the patient must meet before proceeding to the next step of their gender transition. The seven elements to include in a letter of readiness were more explicitly listed within this version as well including: the patient’s identifying characteristics, gender, sexual orientation, any other psychological diagnoses, duration and nature of the treatment with the letter writer, whether the author is part of a gender team, whether eligibility criteria have been met, the patient’s ability to follow the SOC and an offer of collaboration. Version five removes the requirement that patients undertake psychotherapy to be eligible for GAC (37).

#### Results review

Five articles were published between 1998 and 2001 while version five was active. Two of these articles were summaries of the SOC (37, 38). Themes in these publications included continued attempts to develop comprehensive treatment models for GAS.

Ma reviewed the role of the social worker in a multidisciplinary gender clinic in Hong Kong. Psychosocial assessment for GAS included evaluation of performance in affirmed social roles, adaptation to the affirmed gender role during the 1-year RLT and understanding the patient’s identified gender role and the response to the new gender role culturally and interpersonally within the individual’s support network and family unit. She noted five contraindications to GAS: a history of psychosis, sociopathy, severe depression, organic brain dysfunction or “defective intelligence,” success in parental or marital roles, “successful functioning in heterosexual intercourse,” ability to function in the pretransition gender role, and homosexual or TV history with genital pleasure. She proposed a social work practice model for patients who apply for GAS with categorization of TGD individuals into “better-adjusted” and “poorly-adjusted” with different intervention goals and methods for each. For those who were “better-adjusted,” treatment focused on psychoeducation, building coping tools, and mobilization into a peer counselor role, while treatment goals for those who were “poorly-adjusted” focused on building support and resources (39).

Damodaran and Kennedy reviewed the assessment and treatment model used by the Monash gender dysphoria clinic in Melbourne, Australia for patients requesting GAS. All referrals for GAS were assessed independently by two psychiatrists to determine proper diagnosis of gender dysphoria, followed by endocrinology and psychology consultation to develop a comprehensive treatment plan. Requirements included RLT of minimum 18 months and GAH (40).

Miach reviewed the utility of using the Minnesota Multiphasic Personality Inventory-2 (MMPI-2), a revision of the MMPI which was standardized using a more heterogeneous population, in a gender clinic to assess stability of psychopathology prior to GAS, which was only performed on patients aged 21–55 years old. The authors concluded that while the TGD group had a significantly lower level of psychopathology than the control group, they believed that the MMPI-2 was a useful test in assessing readiness for GAC (41).

### Standards of care version 6

#### Changes to the standards of care

Published in 2001, version six of the WPATH SOC did not include significant changes to the 10 tasks of the mental health professional (Figure 3) or in the general recommendations for content of the letters of readiness. An important change in the eligibility criteria for GAH allowed providers to prescribe hormones even if patients had not undergone RLT or psychotherapy if it was for harm reduction purposes (i.e., to prevent patient from buying black market hormones). A notable change in version six separated the eligibility and readiness criteria for top (breast augmentation or mastectomy) and bottom (any gender-affirming surgical alteration of genitalia or reproductive organs) surgery allowing some patients, particularly individuals assigned female at birth (AFAB), to receive a mastectomy without having been on GAH or completing a 12 month RLT (42, 43).

#### Results review

Thirteen articles were published between 2001 and 2012. One is a systematic review of evidence for factors that are associated with regret and suicide, and predictive factors of a good psychological and social functioning outcome after GAC. De Cuypere and Vercruysse note that less than one percent of patients regret having GAS or commit suicide, making detection of negative predictive factors in a study nearly impossible. They identified a wide array of positive predictive factors including age at time of request, sex of partner, premorbid social or psychiatric functioning, adequacy of social support system, level of satisfaction with secondary sexual characteristics, and surgical outcomes. Many of these predictive factors were later disproved. They also noted that there were not enough studies to determine whether following the WPATH guidelines was a positive predictive factor. In the end they noted that the evidence for all established evaluation regimens (i.e., RLT, age cut-off, psychotherapy, etc.) was at best indeterminate. They recommended that changes to WPATH criteria should redirect focus from gender identity to psychopathology, differential diagnosis, and psychotherapy for severe personality disorders (44).

The literature at this time supports two opposing approaches to requests for GAC, those advocating for a set of strictly enforced eligibility and readiness criteria associated with very thorough evaluations and those who advocate for a more flexible approach. Common approaches to the evaluation for GAC include: taking a detailed social history including current relationships, support systems, income, and social functioning; a sexual development history meant to understand when and how the patient began to identify as TGD and how their transition has affected their life; an evaluation of their coping skills, “psychic functions” and general mental well-being; and a focus on assessing the “correct diagnosis” of gender identity disorder (44–56). The use of a multidisciplinary team was also commonly recommended (44, 47, 48, 51, 54–56).

Those that advocated for a stricter interpretation of the eligibility and readiness criteria emphasized the importance of the RLT (45, 49, 51, 53, 55, 56). One clinic in the UK required a RLT lasting 2 years prior to starting GAH, twice as long as recommended by the SOC (49). The prevailing view continued to approach gender as a binary phenomenon, rather than as a spectrum of experiences. As a result, treatment recommendations emphasized helping the patient to “pass” in their chosen gender role and did not endorse patients receiving less than the full spectrum of treatment to transition fully from one sex to the other. Several authors indicated that they required some amount of psychotherapy before recommending GAC (46, 47, 51, 52, 55, 56). One author described requirements in Turkey, which unlike the US has the requirements enshrined in law and defines an important role for the courts in granting permission for GAC (51). In general, these authors supported the gatekeeping role of the mental health provider as a mechanism to prevent cases of regret.

Among groups supporting a flexible interpretation of the SOC, there was a much stronger emphasis on the supportive role of the mental health provider in the gender transition process (44–46, 48, 52, 53). This role included creating a supportive environment for the patient, asking and using the correct pronouns, and helping to guide them through what may be a difficult transition both socially and physically. They emphasized the importance of the psychosocial evaluation including the patient’s connections to others in the TGD community, their social functioning, substance use, and psychiatric history/psychological functioning. While informed consent was mentioned as part of the evaluation, the process was not thoroughly explored and largely focused on patients’ awareness that GAS is an irreversible procedure which removes healthy tissue (53). One author suggested that a “consumer handbook outlining such rights and responsibilities” related to GAS be made available, but they made no further comment on the informed consent process (44). There was no further guidance as to the contents of letters of readiness for GAC.

The lack of emphasis on informed consent by both groups of authors mirrors the discussion of informed consent within the SOC, which up through version six, had a relatively narrow definition and role specifically related to risks and benefits of surgery. As far back as version one, the SOC states “hormonal and surgical sex reassignment are procedures which must be requested by, and performed only with the agreement of, the patient having informed consent…[these procedures] may be conducted or administered only after the patient applicant has received full and complete explanations, preferably in writing, in words understood by the patient applicant, of all risks inherent in the requested procedures (16). “This reflects the dominant concerns of surgeons at the time that they were removing or damaging healthy tissue, which was unethical, and as such wanted to make sure that patients understood the irreversibility of the procedures. It was not until version 7 that there is a change in the discussion of informed consent.

### Standards of care version 7

#### Changes to the standards of care

Standards of care version seven was published in 2013. Publication of version seven coincided with the publication of DSM-5, in which the diagnosis required to receive GAC shifted from Gender Identity Disorder to Gender Dysphoria, in an effort to de-pathologize TGD patients. Version seven highlights that these are *guidelines* meant to be flexible to account for different practices in different places. Compared to version six, a significantly expanded section on the “Tasks of the Mental Health Provider” was added, offering some instructions on what to include in the assessment of the patient for GAS. For the first time the SOC expand on what it means to obtain informed consent and describe a process where the mental health provider is expected to guide a conversation around gender identity and how different treatments and procedures might affect TGD individuals psychologically, socially, and physically. Other recommendations include “at a minimum, assessment of gender identity and gender dysphoria, history and development of gender dysphoric feelings, the impact of stigma attached to gender non-conformity on mental health, and the availability of support from family, friends, and peers.” There is also a change to the recommended content of the letters: switching from “The initial and evolving gender, sexual, and other psychiatric diagnoses” to “Results of the client’s psychosocial assessment, including any diagnoses”, indicating a shift in the focus away from diagnosis toward the psychosocial assessment. Version 7 also adds two new tasks for the mental health provider including “Educate and advocate on behalf of clients within their community (schools, workplaces, other organizations) and assist clients with making changes in identity documents” and “Provide information and referral for peer support”(2).

There were also significant changes to eligibility criteria for GAC. For GAH, version seven eliminates entirely the requirement for a RLT and psychotherapy and adds requirements for “persistent well documented gender dysphoria” and “reasonably well controlled” medical or mental health concerns. Notably, the SOC do not define the meaning of “reasonably well controlled,” leaving providers to interpret this on their own. Version seven delineates separate requirements for top and bottom surgeries. The criteria for both feminizing and masculinizing top surgeries are identical to each other and identical to those laid out for GAH. Version seven explicitly states that GAH is not required prior to top surgery, although GAH is still recommended prior to gender-affirming breast augmentation. Criteria for bottom surgery are more explicitly defined, namely internal (i.e., hysterectomy, orchiectomy) vs. external (i.e., metoidioplasty, phalloplasty, and vaginoplasty). For internal surgeries, criteria are the same as for top surgery with the addition of a required 12 months of GAH. For external surgeries the criteria are the same as for internal, with the addition of required 12 months of living in the patient’s affirmed gender identity (2, 42).

#### Results review

Twenty-three articles were published while version 7 of the SOC have been active. Themes include identifying the role of psychometric testing in GAC evaluations, expanding the discussion around informed consent for GAC, and revising the requirements for letter writers.

A systematic review evaluated the accuracy of psychometric tests in those requesting GAC, identifying only two published manuscripts that met their inclusion criteria, both of which were of poor quality; this led them to question the utility of psychometric tests in in TGD patients (57). Keo-Meir and Fitzgerald provided a detailed narrative review of psychometric and neurocognitive exams in the TGD population and concluded that psychometric testing should not be done unless there is a question about the capacity of the patient to provide informed consent (58). The only other manuscripts that include a mention of psychological testing describe processes in Iran and China, both of which require extensive psychological testing prior to approval for GAC (59, 60). These two manuscripts, in addition to an ethnographic study of the evaluation process in Turkey (61), are also the only ones that indicate a requirement for psychotherapy prior to approval for treatment. The three international manuscripts described above plus three manuscripts from the US (62–64) are the only ones to include consideration of a RLT, with authors outside the US preferring a long RLT and US authors considering RLT as part of the informed consent process for GAS, and not required at all prior to the initiation of GAH.

Many authors describe the process of informed consent for GAC (1, 58, 60, 62–76). In China, a signature indicating informed consent from the patient’s family is required in addition to that of the patient (60). Many authors emphasize evaluating for and addressing social determinants of health including housing status, income, transportation, trauma history, etc. (1, 58, 60, 67, 69–71, 75–77). Deutsch advocated for the psychosocial evaluation being the most important aspect of the evaluation and suggests that one of the letters required for bottom surgery be replaced by a functional assessment (i.e., ADLs/iADLs), which could be repeated as needed or removed entirely for high functioning patients (69).

Practice patterns and opinions on who should write letters of readiness and how many letters should be required vary widely. Many letters that surgeons receive are cursory, and short and non-personal letters correlate with poor surgical outcomes (1). Several authors advocate for eliminating the second letter entirely, for at least some procedures, as it is a barrier to care (68, 69, 74). Some support removing the requirement that both letter writers be therapists or psychiatrists, and even suggesting the second letter be written by a urologist (72) or a social worker who has performed a detailed social assessment (69, 75). The evaluation in Turkey requires a report written by an extensive multidisciplinary team and submitted to a court for approval (61). Surveys of providers indicate that the SOC are not uniformly implemented leading to huge disparities based on the providers knowledge level and personal beliefs (77, 78). Additional recommendations include that providers spend significant time discussing the SOC and diagnosis of gender dysphoria with the patients prior to providing a letter to prepare them for the stigma such a diagnosis may confer (65, 66), and dropping gender dysphoria entirely in favor the ICD-11 diagnosis of gender incongruence, as it may be less stigmatizing (71).

The Mount Sinai Gender Clinic describes an integrated multidisciplinary model where a patient will see a primary care doctor, endocrinologist, social worker, psychiatrist, and obtain any necessary lab work in a single visit, significantly reducing barriers to care. The criteria in this model focus on informed consent, the social determinants of health, being physically ready for surgery, and putting measurable goals on psychiatric stability, while deemphasizing the gender dysphoria diagnosis. Their study showed that people who received their evaluation over a 2-year period were more likely to meet their in-house criteria than they were to meet criteria as set forth in WPATH SOC. The Mount Sinai criteria allowed for significantly decreased barriers to care, allowing more people to progress through desired GAC in a timely fashion (75).

### Standards of care version 8

#### Changes to the standards of care

Standards of care version 8, published in September 2022, includes major updates to the guidelines around GAS. This version explicitly highlights the importance of informed decision making, patient autonomy, and harm reduction models of care, as well as emphasizing the flexibility of the guidelines which the authors note can be modified by the healthcare provider in consultation with the TGD individual.

Version 8 lays out the roles of the assessor which are to identify the presence of gender incongruence and any co-existing mental health concerns, provide information on GAC, support the TGD individual in their decision-making, and to assess for capacity to consent to GAC. The authors emphasize the collaborative nature of this decision-making process between the assessor and the TGD individual, as well as recommending TGD care occur in a multidisciplinary team model when possible.

Version 8 recommends that providers who assess TGD individuals for GAC hold at least a Master’s level degree and have sufficient knowledge in diagnosing gender incongruence and distinguishing it from other diagnoses which may present similarly. These changes allow for non-mental health providers to be the main assessors for GAC.

Version 8 recommends reducing the number of evaluations prior to GAS to a single evaluation in an effort to reduce barriers to care for the TGD population. Notably, the authors have removed the recommendations around content of the letter of readiness for GAC. The guidelines note that the complexity of the assessment process may differ from patient to patient, based on the type of GAC requested and the specific characteristics of the patient. Version eight directly states that psychometric testing and psychotherapy are not requirements to pursue GAC. While evaluations should continue to identify co-existing mental health diagnoses, version 8 highlights that the presence of a mental health diagnosis should not prevent access to GAC unless the mental health symptoms directly interfere with capacity to provide informed consent for treatment or interfere with receiving treatment. Version 8 recommends that perioperative matters, such as travel requirements, presence of stable, safe housing, hygiene/healthy living, any activity restrictions, and aftercare optimization, be discussed by the surgeon prior to GAS. In terms of eligibility criteria, the authors recommend a reduced duration of GAH from 12 months (from version 7) to 6 months (in version 8) prior to pursuing GAS involving reproductive organs (79).

### Ethical discussions

#### Results review

A total of eleven articles explored ethical considerations of conducting mental health evaluations and writing letters of readiness for GAS, including a comparison of the ethical principles prioritized within the “gatekeeping” model vs. the informed consent model for GAC and the differential treatment of TGD individuals compared to cisgender individuals seeking similar surgical procedures.

Many authors compare the informed consent model of care for TGD individuals to the WPATH SOC model. In the informed consent model, the role of the health practitioner is to provide TGD patients with information about risks, side effects, benefits, and possible consequences of undergoing GAC, and to obtain informed consent from the patient (80). Cavanaugh et al. argue that the informed consent model is more patient-centered and elevates the ethical principle of autonomy above non-maleficence, the principle often prioritized in the “gatekeeping” model (81). They write, “Through a discussion of risks and benefits of possible treatment options with the patient…clinicians work to assist patients in making decisions. This approach recognizes that patients are the only ones who are best positioned, in the context of their lived experience, to assess and judge beneficence (i.e., the potential improvement in their welfare that might be achieved), and it also affords prescribing clinicians a better and fuller sense of how a particular patient balances principles of non-maleficence and beneficence.” Authors note that mental health providers can be particularly helpful in situations where an individual desires additional mental health treatment, which some argue should remain optional, or when an individual’s capacity is in question (81). Additional ethical considerations include balancing the respect for the dignity of persons, responsible caring, integrity in relationships, and responsibility to society (82). Other authors argue for a more systematic approach to ethical issues, including consulting the literature and/or experts in the field of TGD mental health for support in making decisions around GAC (74).

Hale criticizes the WPATH SOC noting that these guidelines create a barrier between patient and mental health provider in establishing trust and a therapeutic relationship, overly pathologize TGD individuals, and unnecessarily impose financial costs to the TGD individual. As a “gatekeeper,” the mental health provider is placed in the position of either granting or denying GAC and must weigh the competing ethical principles of beneficence, non-maleficence, and autonomy. He argues that mental health providers are not surrogate decision makers and that framing requests for GAS as a “phenomenon of incapacity” is “reflective of the overall incapacitating effects of society at large toward the TGD community” (83). This reflects the broader approach to determining capacity utilized in other medical contexts, namely that patients have capacity until proven otherwise (84). Additionally, due to the gatekeeping dynamic between patient and clinician, many TGD patients may not mention concerns or fears surrounding GAS out of concern they will be denied services, thereby limiting the quality and utility of the informed consent discussion. Ashley proposes changes to the informed consent model, specifically that the informed consent process should include not only information about whether to go through with a procedure, but how to go through the procedure including relevant information about timeline, side effects, need for perioperative support, and treatment plan (85). Gruenweld argues for a bottom-up, TGD-led provision of GAC instead of focusing solely on alleviating gender dysphoria through a top-down, medical expert approach *via* such systems like the WPATH SOC (86).

MacKinnon et al. conducted an institutional ethnographic study of both TGD individuals undergoing mental health evaluations for GAC and mental health providers to better understand the process of conducting such evaluations (87). They found that providers cited three concerns with the evaluation: determining the authenticity of an individual’s TGD identity, determining if the individual has the capacity to consent to treatment, and determining the readiness of the individual to undergo treatment. TGD individuals cited concerns around presenting enough distress to be diagnosed with gender dysphoria (a SOC requirement) versus too much distress, and risk being diagnosed with an uncontrolled mental health condition therefore being ineligible for GAC. The authors conclude, “although they are designed to optimize and universalize care… psychosocial readiness assessments actually create a medically risky and arguably unethical situation in which trans people experiencing mental health issues have to decide what is more important – transitioning at the potential expense of care for their mental health or disclosing significant mental health issues at the expense of being rendered not ready to transition (which in turn may produce or exacerbate mental distress)” (87).

With regards to writing letters of readiness for GAS, authors comment on the differential treatment of TGD compared to cisgender individuals. Bouman argues that requiring two letters for gender-affirming orchiectomy or hysterectomy is unethical given that orchiectomy and hysterectomy for chronic scrotal pain and dysfunctional uterine bleeding, respectively, do not require any mental health evaluation. Requiring a second letter may cause delays in treatment, increase financial costs, and may be invasive to the patient who must undergo two detailed evaluations, while allowing for diffusion of responsibility for the mental health provider (88).

## Discussion

### Changing standards

Starting in the 1950’s with the first successful gender affirming procedure in the US on Christine Jorgenson, TGD people in the US started seeking surgical treatment of what was then called TS. The medical community’s understanding of TGD people, their mental health, and the role of the mental health provider in their medical and surgical transition has progressed and evolved since this time. Prior to the first iteration of what would later be known as WPATH’s SOC, patients were mostly evaluated within a system that viewed gender and sexual minorities as deviants and thereby largely limited access to GAC. We can also see this reflected in the changes to DSM and ICD diagnostic criteria between 1980 and today which demonstrates a trend from pathologizing identity and conflating sexual and gender identity toward pathologizing the distress experienced due to the discordant identity, and finally removing the relevant diagnosis from the chapter of Mental and Behavioral Disorders altogether in the ICD and instead into a new chapter titled “conditions related to sexual health (89).” These changes have clearly yielded positive benefits for TGD individuals by reducing stigma and improving access to care, but significant problems remain. Requiring TGD people to have a diagnosis at all to obtain care, no matter the terminology used, is pathologizing. The practice of requiring a diagnosis continues to put mental health and other medical providers in the position of gatekeeping, continuing the vestigial historical focus on “confirming” a person’s gender identity, rather than trusting that TGD people understand their identities better than providers do. Version 8 of the SOC put a much heavier emphasis on shared decision making and informed consent, but continue to maintain the requirement of a diagnosis (79). Many insurance companies and other health care payers require the diagnosis to justify paying for GAC, but providers should continue to advocate for removing such labels as a gatekeeping mechanism for GAC.

With each version of the SOC, guidelines for GAC become more specific, with more explanation of the reasoning behind each recommendation; more flexible requirements, a broadening of the definition of mental health provider, and elimination of the requirement that at least one letter be written by a doctoral level provider. There has been a notable shift in the conceptualization of gender identity, away from a strict gender binary, with individuals transitioning fully from one end to the other, to gender identity and transition as a spectrum of experiences. Over time the SOC became more flexible by removing requirements for psychotherapy, narrowing requirement for the RLT to only those pursuing bottom surgery, eliminating requirements for a mental health evaluation prior to initiating GAH, and eliminating requirements for GAH prior to top surgery. Version 8 of the SOC was even more explicit about removing requirements for psychotherapy and psychometric testing prior to receiving GAC (79).

Despite these positive changes, those wishing to access GAC still face significant challenges. Access to providers knowledgeable about GAC remains limited, especially in more rural areas, therefore requiring evaluations and letters of readiness for GAC continues to significantly limit access to treatment. By requiring letters of readiness for GAC, adult TGD individuals are not afforded the same level of autonomy present in almost any other medical context, where capacity to provide informed consent is automatically established (84). The WPATH SOC continue to perpetuate differential treatment of TGD individuals by requiring extensive, and often invasive, evaluations for procedures that their cisgender peers are able to access without such evaluations (88). The WPATH guidelines apply a one-size-fits-all approach to an extremely heterogeneous community who have varying levels of needs based on a variety of factors including but not limited to age, socioeconomic status, race, natal sex, and geographic location (90). It should be noted, however, that the version 8 of the SOC does acknowledge that different patients may require evaluations of varying complexity based on the procedure they are requesting as well as a variety of psychosocial factors, although it remains vague about exactly what those different evaluations should entail (79). We propose that future work be directed toward three primary goals: conducting research to determine the utility of letters of readiness; to better understand factors that impact GAS outcomes; and to develop easily accessible and understandable guides to conducting readiness evaluations and writing letters. These aims will help to further our goals of advocating for this vastly underserved population by further removing barriers to life-saving GAC.

### Changing ethics

Early iterations of the SOC were strict, placing the mental health provider within a gatekeeper role, tasked with distinguishing the “true transsexual” that would benefit from GAS from those who would not, which in effect elevated the ethical principal of non-maleficence above autonomy. This created a barrier to forming a therapeutic alliance between the patient and mental health provider as there was little motivation for patients to give any information outside of the expected gender narrative (50, 65). Mistrust flowed both ways leading to longer and more involved evaluations then than what is required today, with many providers requiring patients to undergo extensive psychological testing and psychotherapy, provide extensive collateral, and undergo lengthy RLTs, with some focusing on a patient’s ability to “pass” within the desire gender role, before agreeing to write a letter (11, 15, 19, 49, 57, 58).

As understanding around the experiences of TGD individuals has evolved over time, the emphasis has shifted from the reliance on non-maleficence toward elevating patient autonomy as the guiding principle of care. Evaluations within this informed consent model focus much more on the patient’s ability to understand the treatment, its aftercare, and its potential effect on their lives. Informed consent evaluations also shift focus toward other psychosocial factors that will contribute to successful surgical outcomes, for example, housing, transportation, a support system, and treatment of any underlying mental health symptoms. While there is still a lack of consistency in current evaluations and the SOC are enforced unevenly (77), the use of the informed consent model by some providers has reduced barriers for some patients. Many authors now agree that psychological or neuropsychological testing should not be used when evaluating for surgical readiness unless there is a concern about the patient’s ability to provide informed consent such as in the case of a neurocognitive or developmental disorder (58). Also important to note here is that while there is a general shift in the focus of the literature from that of gatekeeping toward one of informed consent, neither the informed consent model nor the WPATH SOC more broadly are evenly applied by providers, leading to continued barriers for many patients (77, 78).

Within the literature, there is support for further reducing barriers to care by widening the definition of who can conduct evaluations, write letters, or facilitate the informed consent discussion for GAC. Recommending that the physician providing the GAC be the one to conduct the informed consent evaluation would bring GAC practices more in line with practices in place within the broader medical community. It is very rare for mental health providers to be the gatekeepers for medical or surgical procedures, except for transplant surgery, where mental health providers may have a clearer role given the prominence of substance use disorders and the very limited resource of organs. However, even within transplant psychiatry, a negative psychiatric evaluation would not necessarily preclude the patient from receiving the transplant, but instead may be used to guide a treatment plan to improve chances of a successful recovery post-operatively. We then should consider what it means to embrace patient autonomy as our guiding principle, especially with more than 40 years of evidence of the positive effects around GAC behind us. Future guidelines should focus on making sure that TGD individuals are good surgical candidates, not based on their gender identity, but instead on a more holistic understanding of the factors that lead to good and bad gender-affirming surgical outcomes, along the lines of those proposed by Mt. Sinai’s gender clinic for vaginoplasty (75). Additionally, the physicians providing the GAC should in most cases be the ones to obtain informed consent, while retaining the ability to request a mental health evaluation if specific concerns related to mental health arise. This would both allow mental health providers to adopt a supportive consultant role rather than that of gatekeeper, as well as provide more individualized rather than one-size-fits-all care to patients.

Version 8 of the SOC go a long way toward changing the ethical focus of evaluations toward one of shared decision making and informed consent by removing the requirement of a second letter and the requirement that the letter be written by a mental health provider. This will, in theory, lower barriers to care by allowing other providers (as long as they have at least a master’s degree) to write letters for surgery (79). In practice, however, this change is likely to only affect a small portion of the patient population. This is because, as noted in the section below in more detail, insurance companies already do not adhere closely to the SOC (91) and are unlikely to quickly adopt the new guidelines if at all. Further, it is possible that many surgeons will require that the letter of readiness be written by a mental health provider, especially if the patient has any previous mental health problems. While changes to SOC 8 are a step in the direction we propose in this manuscript, it is important to remember that the primary decision makers of who can access GAC in the US are insurance companies with surgeons, primary care providers, and mental health providers as secondary decision makers; this leaves patients with much less real-world autonomy than the SOC state they should have in the process. While insurance companies hold this effective decision-making power in all of US healthcare, it could be at least partially addressed by developing clear, evidence based guidelines for which patients might require a more in-depth evaluation in the first place. Screening out patients that have little or no mental health or social barriers to care would directly reduce those patients’ barriers to receiving GAC, while freeing up mental health and other providers to provide evaluation, resources, and support to those patients who will actually benefit from these services.

### Letter writing

There are few published guides for writing letters of readiness for GAC. The WPATH SOC provide vague guidelines as to the information to include within the letter itself, which, in addition to a lack of consistency in implementation of the SOC, lead to a huge variety in current practices around letter writing and limit their usefulness to surgical providers (1). There is much debate within the literature about how many letters should be required and who should be able to write them. Guidelines from China, Turkey, and Iran recommend much stricter processes requiring input from a wider variety of specialists to comment on a patient’s readiness (59–61). Within the US, the few recent recommendations include having a frank discussion with patients about the gender dysphoria diagnosis and allowing them to have input into the content of the letter itself (65, 66, 70, 71, 75). The heterogeneity of current practices around letter writing demonstrates a reality in which many providers do not uniformly operate within the informed consent model, and do not even uniformly adhere to the SOC as written. This heterogeneity in practice by providers also extends to requirements by insurance companies in the US. The lack of clear guidelines about what should go into a letter, especially across different insurance providers, can lead to increased barriers to care due to insurance denials for incorrectly written letters. While direct data examining insurance denials for incorrectly written letters is not available, we can see this indirect effects in the fact that while 90% of insurance providers in the US provide coverage for GAC, only 5–10% of TGD patients had received bottom surgery even though about 50% of TGD patients have reported wanting it (91). Version 8 of the SOC reduce some of the letter writing requirements as discussed above, but they still do not give clear instructions on exactly how to write a letter of readiness or perform an evaluation (79). Given the lack of uniformity and limited benefit of such letters to surgical providers, these authors propose that future research be conducted into the need for letters of readiness for GAC, ways to ensure the content of such letters are evidence-based to improve outcomes of GAC, and improve education to providers by creating an easily accessible and free semi-structured interview with letter template.

## Limitations

The reviewed articles included opinion manuscripts, published SOC, and proposed models for how to design and operate GAC clinics, however, this narrative review is limited by a lack of peer reviewed clinical trials that assess the evidence for the GAC practices described here. As a result, it is challenging to comment on the effectiveness of various interventions over time.

## Conclusion

The WPATH SOC have evolved significantly over time with regards to their treatment of TGD individuals. Review of the literature shows a clear progression of practices from paternalistic gatekeeping toward increasing emphasis on patient autonomy and informed consent. Mental health evaluations, still required by SOC version eight are almost entirely unique as a requirement for GAS, apart from some bariatric and transplant surgeries. Individuals who wish to pursue GAC are required to get approval for treatments that their cisgender peers may pursue without such evaluations. While there may be some benefits from these evaluations in helping to optimize a patient socially, emotionally, and psychologically for GAC, the increased stigma and burden placed on patients by having a blanket requirement for such evaluations leads us to seriously question the readiness evaluation requirements in SOC version 8, despite a reduction in the requirements compared to previous SOC. This burden is made worse by limited access to providers knowledgeable and competent in conducting GAC evaluations, writing letters of readiness, and a lack of consistency in the application and interpretations of the SOC by both providers and insurance companies. Other barriers to care created by multiple letter requirements include the often-prohibitive cost of getting multiple evaluations and the delay in receiving their medical or surgical treatments due to extensive wait times to see a mental health provider. This barrier will in theory be ameliorated by updates to SOC in version 8, but multiple letters are likely to at least be required by insurance companies for some time. Overall, the shift from gate keeping to informed consent has been a net positive for patients by reducing barriers to care and improving patient autonomy, but the mental health evaluation is still an unnecessary barrier for many people. Further research is necessary to develop a standardized evaluation and letter template for providers to access, as well as further study into who can most benefit from an evaluation in the first place.

## Data availability statement

The original contributions presented in this study are included in the article/Supplementary material, further inquiries can be directed to the corresponding author.

## Author contributions

TA and KK contributed to the conception and design of the study under the guidance of RL and AJ, reviewed and analyzed the literature, and wrote the manuscript. AW organized the literature search and wrote the “Methods” section. RL and AJ assisted in review and revision of the completed manuscript. All authors approved of the submitted version.

## Footnotes

1 Gender affirming surgery has historically been referred to as sexual reassignment surgery (SRS).

2 Gender affirming care is an umbrella term referring to any medical care a TGD individual might pursue that affirms their gender identity, including primary care, mental health care, GAH or GAS.

3 The organization will be referred to as WPATH moving forward, even when referring to time periods before the name change.

## Abbreviations

TGD: transgender and gender diverse
SRS: sex reassignment surgery
WPATH: World Professional Association for Transgender Health
SOC: standards of care
GAC: gender-affirming care
GAS: gender-affirming surgery
GAH: gender-affirming hormones
TS: transsexualism
TV: transvestism
HBIGDA: Harry Benjamin International Gender Dysphoria Association
RLT: Real life test
MMPI: Minnesota Multiphasic Personality Inventory
FTM: female to male
MTF: male to female
LGBTQI: lesbian, gay, bisexual, transgender, queer, intersex
DSM: diagnostic and statistical manual of mental disorders
ICD: international classification of diseases.
