# Supplementary material for: Readiness assessments for gender-affirming surgical treatments: A systematic scoping review of historical practices and changing ethical considerations
Source: Front Psychiatry. 2022 Oct 20;13:1006024. doi: 10.3389/fpsyt.2022.1006024 (PMC9630738; doi:10.3389/fpsyt.2022.1006024)
Supplement: Supplementary file 1 [file Table_1.DOCX]

| **Database searched** | **Date searched** | **Results** |
| --- | --- | --- |
| MEDLINE (PubMed) | 12/02/2020 | 1609 |
| Cochrane Database of Systematic Reviews  Issue 10 of 12, October 2019 (Wiley) | 12/02/2020 | 23 |
| Cochrane Central Register of Controlled Trials  Issue 10 of 12, October 2019 (Wiley) | 12/02/2020 | 1899 |
| CINAHL Plus with Full Text (Ebsco) | 12/02/2020 | 997 |
| PsycINFO (Ebsco) | 12/02/2020 | 1394 |
| Scopus (Elsevier) | 12/02/2020 | 2182 |
| ProQuest Dissertations & Theses Global | 12/02/2020 | 93 |
| Total | | 8197 |
| After de-duplication | | **4411** |

| **MEDLINE (PubMed)** | | |
| --- | --- | --- |
| **#** | **Search** | **Result** |
| 1 | ("Mental Health"[Mesh] OR "Psychiatry"[Mesh] OR "Psychology"[Mesh] OR "Psychological Tests"[Mesh] OR “Sexology”[Mesh] OR "psychology" [Subheading] OR “mental health”[tiab] OR “mental hygiene”[tiab] OR “mental status”[tiab] OR “mental care”[tiab] OR “mental condition”[tiab] OR psychosocial[tiab] OR psycholog*[tiab] OR psychiatr*[tiab] OR psychotherap*[tiab] OR neuropsych*[tiab] OR neurocognitive[tiab] OR psychometric*[tiab] OR sexolog*[tiab])  AND  ((("Transgender Persons"[Mesh] OR "Health Services for Transgender Persons"[Mesh] OR "Gender Dysphoria"[Mesh] OR "Transsexualism"[Mesh] OR “transgender*”[tiab] OR “transsex*”[tiab] OR “trans patient*”[tiab] OR “trans person*”[tiab] OR “trans people”[tiab] OR “gender non-conforming”[tiab] OR “non-binary”[tiab] OR “nonbinary”[tiab] OR “gender dysphoria”[tiab] OR “gender identity disorder*”[tiab] OR “genderqueer”[tiab] OR “gender incongru*”[tiab] OR “male to female”[tiab] OR “female to male”[tiab] OR FTM[tiab] OR MTF[tiab] OR transmasculine[tiab] OR transfeminine[tiab]) AND ( "Surgical Procedures, Operative"[Mesh] OR "surgery"[Subheading] OR surg*[tiab] OR operat*[tiab] OR presurg*[tiab] OR preoperat*[tiab] OR Vaginoplast*[tiab] OR Phalloplast*[tiab] OR “chest reconstruction”[tiab] OR “breast reconstruction”[tiab] OR “chest masculinization”[tiab] OR Mastectom*[tiab] OR “breast augmentation”[tiab] OR Orchiectom*[tiab] OR orchidectomy*[tiab] OR clitoroplast*[tiab] OR labiaplast*[tiab] OR Hysterectom*[tiab] OR Oophorectom*[tiab] OR Penectom*[tiab] OR scrotoplast*[tiab] OR urethroplast*[tiab] OR vaginectom*[tiab])) OR ("Sex Reassignment Surgery"[Mesh] OR “gender affirm*”[tiab] OR “gender reaffirm*”[tiab] OR “sex affirm*”[tiab] OR “gender confirm*”[tiab] OR “sex confirm*”[tiab] OR “gender reassignment*”[tiab] OR “sex reassignment*”[tiab] OR “genital reassignment*”[tiab] OR “sex change*”[tiab] OR “medical transition*”[tiab])) | **1584** |

| **Cochrane Library** | | |
| --- | --- | --- |
| **#** | **Search** | **Result** |
| #1 | MeSH descriptor: [Mental Health] explode all trees | 1489 |
| #2 | MeSH descriptor: [Psychiatry] explode all trees | 470 |
| #3 | MeSH descriptor: [Psychology] explode all trees | 1052 |
| #4 | MeSH descriptor: [Psychological Tests] explode all trees | 22067 |
| #5 | MeSH descriptor: [Sexology] explode all trees | 280 |
| #6 | Any MeSH descriptor in all MeSH products and with qualifier(s): [psychology - PX] | 56743 |
| #7 | (“mental health” OR “mental hygiene” OR “mental status” OR “mental care” OR “mental condition” OR psychosocial OR psycholog* OR psychiatr* OR psychotherap* OR neuropsych* OR neurocognitive OR psychometric OR sexolog*):ti,ab,kw | 152460 |
| #8 | #1 OR #2 OR #3 OR #4 OR #5 OR #6 OR #7 | 153469 |
| #9 | MeSH descriptor: [Transgender Persons] explode all trees | 40 |
| #10 | MeSH descriptor: [Health Services for Transgender Persons] explode all trees | 0 |
| #11 | MeSH descriptor: [Gender Dysphoria] explode all trees | 4 |
| #12 | MeSH descriptor: [Transsexualism] explode all trees | 27 |
| #13 | (transgender* OR transsex* OR “trans patient” OR “trans patients” OR “trans person” OR “trans persons” OR “trans people” OR “gender non-conforming” OR “non-binary” OR “nonbinary” OR “gender dysphoria” OR “gender identity disorder” OR “gender identity disorders” OR “genderqueer” OR “gender incongruence” OR “gender incongruences” OR “gender incongruencies” OR “gender incongruity” OR “gender incongruities” OR “male to female” OR “female to male” OR FTM OR MTF OR transmasculine OR transfeminine):ti,ab,kw | 965 |
| #14 | #9 OR #10 OR #11 OR #12 OR #13 | 965 |
| #15 | MeSH descriptor: [Surgical Procedures, Operative] explode all trees | 118224 |
| #16 | Any MeSH descriptor in all MeSH products and with qualifier(s): [surgery - SU] | 57971 |
| #17 | (surg* OR operat* OR presurg* OR preoperat* OR Vaginoplast* OR Phalloplast* OR “chest reconstruction” OR “breast reconstruction” OR “chest masculinization” OR Mastectom* OR “breast augmentation” OR Orchiectom* OR orchidectomy* OR clitoroplast* OR labiaplast* OR Hysterectom* OR Oophorectom* OR Penectom* OR scrotoplast* OR urethroplast* OR vaginectom*):ti,ab,kw | 286244 |
| #18 | #15 OR #16 OR #17 | 324188 |
| #19 | #14 AND #18 | 304 |
| #20 | MeSH descriptor: [Sex Reassignment Surgery] explode all trees | 2 |
| #21 | (“gender affirm” OR “gender affirmation” OR “gender affirming” OR “gender reaffirm” OR “gender reaffirmation” OR “gender reaffirming” OR “sex affirm” OR “sex affirmation” OR “sex affirming” OR “gender confirm” OR “gender confirmation” OR “gender confirming” OR “sex confirm” OR “sex confirming” OR “sex confirmation” OR “gender reassignment” OR “sex reassignment” OR “genital reassignment” OR “gender reassignments” OR “sex reassignments” OR “genital reassignments” OR “sex change” OR “sex changes” OR “medical transition” OR “medical transitions”):ti,ab,kw | 54 |
| #22 | #20 OR #21 | 54 |
| #23 | #19 OR #22 | 347 |
| #24 | #8 AND #23 | **23** |

| **Embase** | | |
| --- | --- | --- |
| **#** | **Search** | **Result** |
| 1 | ('mental health'/exp OR 'psychiatry'/exp OR 'psychology'/exp OR 'psychologic test'/exp OR 'sexology'/exp OR 'mental health':ti,ab OR 'mental hygiene':ti,ab OR 'mental status':ti,ab OR 'mental care':ti,ab OR 'mental condition':ti,ab OR psychosocial:ti,ab OR psycholog*:ti,ab OR psychiatr*:ti,ab OR psychotherap*:ti,ab OR neuropsych*:ti,ab OR neurocognitive:ti,ab OR psychometric*:ti,ab OR sexolog*:ti,ab) AND (('transgender'/exp OR 'gender dysphoria'/exp OR 'transsexualism'/exp OR 'transsexuality'/exp OR 'transgender*':ti,ab OR 'transsex*':ti,ab OR 'trans patient*':ti,ab OR 'trans person*':ti,ab OR 'trans people':ti,ab OR 'gender non-conforming':ti,ab OR 'non-binary':ti,ab OR 'nonbinary':ti,ab OR 'gender dysphoria':ti,ab OR 'gender identity disorder*':ti,ab OR 'genderqueer':ti,ab OR 'gender incongru*':ti,ab OR 'male to female':ti,ab OR 'female to male':ti,ab OR ftm:ti,ab OR mtf:ti,ab OR transmasculine:ti,ab OR transfeminine:ti,ab) AND ('surgery'/exp OR 'surgery'/lnk OR surg*:ti,ab OR operat*:ti,ab OR presurg*:ti,ab OR preoperat*:ti,ab OR vaginoplast*:ti,ab OR phalloplast*:ti,ab OR 'chest reconstruction':ti,ab OR 'breast reconstruction':ti,ab OR 'chest masculinization':ti,ab OR mastectom*:ti,ab OR 'breast augmentation':ti,ab OR orchiectom*:ti,ab OR orchidectomy*:ti,ab OR clitoroplast*:ti,ab OR labiaplast*:ti,ab OR hysterectom*:ti,ab OR oophorectom*:ti,ab OR penectom*:ti,ab OR scrotoplast*:ti,ab OR urethroplast*:ti,ab OR vaginectom*:ti,ab) OR 'sex reassignment'/exp OR 'gender affirming surgery'/exp OR 'gender affirm*':ti,ab OR 'gender reaffirm*':ti,ab OR 'sex affirm*':ti,ab OR 'gender confirm*':ti,ab OR 'sex confirm*':ti,ab OR 'gender reassignment*':ti,ab OR 'sex reassignment*':ti,ab OR 'genital reassignment*':ti,ab OR 'sex change*':ti,ab OR 'medical transition*':ti,ab) | **1899** |

| **CINAHL** | | |
| --- | --- | --- |
| **#** | **Search** | **Result** |
|  |  |  |
| #1 | (MH "Mental Health") | 39802 |
| #2 | (MH "Psychiatry+") | 18021 |
| #3 | (MH "Psychology+") | 25260 |
| #4 | (MH "Psychological Tests+") | 163279 |
| #5 | (MH "Diagnosis, Psychosocial+") | 31921 |
| #6 | MW “PF” | 463575 |
| #7 | TI ( “mental health” OR “mental hygiene” OR “mental status” OR “mental care” OR “mental condition” OR psychosocial OR psycholog* OR psychiatr* OR psychotherap* OR neuropsych* OR neurocognitive OR psychometric OR sexolog* ) OR AB ( “mental health” OR “mental hygiene” OR “mental status” OR “mental care” OR “mental condition” OR psychosocial OR psycholog* OR psychiatr* OR psychotherap* OR neuropsych* OR neurocognitive OR psychometric OR sexolog* ) | 367318 |
| #8 | S1 OR S2 OR S3 OR S4 OR S5 OR S6 OR S7 | 835322 |
| #9 | (MH "Transgender Persons+") | 3,020 |
| #10 | (MH "Gender Dysphoria") | 219 |
| #11 | (MH "Transsexualism") | 1,065 |
| #12 | TI ( transgender* OR transsex* OR “trans patient” OR “trans patients” OR “trans person” OR “trans persons” OR “trans people” OR “gender non-conforming” OR “non-binary” OR “nonbinary” OR “gender dysphoria” OR “gender identity disorder” OR “gender identity disorders” OR “genderqueer” OR “gender incongruence” OR “gender incongruences” OR “gender incongruencies” OR “gender incongruity” OR “gender incongruities” OR “male to female” OR “female to male” OR FTM OR MTF OR transmasculine OR transfeminine ) OR AB ( transgender* OR transsex* OR “trans patient” OR “trans patients” OR “trans person” OR “trans persons” OR “trans people” OR “gender non-conforming” OR “non-binary” OR “nonbinary” OR “gender dysphoria” OR “gender identity disorder” OR “gender identity disorders” OR “genderqueer” OR “gender incongruence” OR “gender incongruences” OR “gender incongruencies” OR “gender incongruity” OR “gender incongruities” OR “male to female” OR “female to male” OR FTM OR MTF OR transmasculine OR transfeminine ) | 29,059 |
| #13 | S9 OR S10 OR S11 OR S12 | 30028 |
| #14 | (MH "Surgery, Operative+") | 672608 |
| #15 | MW "su" | 374022 |
| #16 | TI ( surg* OR operat* OR presurg* OR preoperat* OR Vaginoplast* OR Phalloplast* OR “chest reconstruction” OR “breast reconstruction” OR “chest masculinization” OR Mastectom* OR “breast augmentation” OR Orchiectom* OR orchidectomy* OR clitoroplast* OR labiaplast* OR Hysterectom* OR Oophorectom* OR Penectom* OR scrotoplast* OR urethroplast* OR vaginectom* ) OR AB ( surg* OR operat* OR presurg* OR preoperat* OR Vaginoplast* OR Phalloplast* OR “chest reconstruction” OR “breast reconstruction” OR “chest masculinization” OR Mastectom* OR “breast augmentation” OR Orchiectom* OR orchidectomy* OR clitoroplast* OR labiaplast* OR Hysterectom* OR Oophorectom* OR Penectom* OR scrotoplast* OR urethroplast* OR vaginectom* ) | 555001 |
| #17 | S14 OR S15 OR S16 | 1030460 |
| #18 | S13 AND S17 | 4655 |
| #19 | (MH "Gender Affirmation Procedures+") | 378 |
| #20 | TI ( “gender affirm” OR “gender affirmation” OR “gender affirming” OR “gender reaffirm” OR “gender reaffirmation” OR “gender reaffirming” OR “sex affirm” OR “sex affirmation” OR “sex affirming” OR “gender confirm” OR “gender confirmation” OR “gender confirming” OR “sex confirm” OR “sex confirming” OR “sex confirmation” OR “gender reassignment” OR “sex reassignment” OR “genital reassignment” OR “gender reassignments” OR “sex reassignments” OR “genital reassignments” OR “sex change” OR “sex changes” OR “medical transition” OR “medical transitions” ) OR AB ( “gender affirm” OR “gender affirmation” OR “gender affirming” OR “gender reaffirm” OR “gender reaffirmation” OR “gender reaffirming” OR “sex affirm” OR “sex affirmation” OR “sex affirming” OR “gender confirm” OR “gender confirmation” OR “gender confirming” OR “sex confirm” OR “sex confirming” OR “sex confirmation” OR “gender reassignment” OR “sex reassignment” OR “genital reassignment” OR “gender reassignments” OR “sex reassignments” OR “genital reassignments” OR “sex change” OR “sex changes” OR “medical transition” OR “medical transitions” ) | 753 |
| #21 | S19 OR S20 | 967 |
| #22 | S18 OR S21 | 5149 |
| #23 | S8 AND S22 | **997** |

| **PsycINFO** | | |
| --- | --- | --- |
| **#** | **Search** | **Result** |
|  |  |  |
| #1 | DE "Mental Health" OR DE "Mental Status" | 75639 |
| #2 | DE "Psychiatry" OR DE "Adolescent Psychiatry" OR DE "Biological Psychiatry" OR DE "Child Psychiatry" OR DE "Community Psychiatry" OR DE "Consultation Liaison Psychiatry" OR DE "Forensic Psychiatry" OR DE "Geriatric Psychiatry" OR DE "Military Psychiatry" OR DE "Neuropsychiatry" OR DE "Orthopsychiatry" OR DE "Social Psychiatry" OR DE "Telepsychiatry" OR DE "Transcultural Psychiatry" | 65350 |
| #3 | DE "Psychology" OR DE "Abnormal Psychology" OR DE "Applied Psychology" OR DE "Clinical Psychology" OR DE "Cognitive Psychology" OR DE "Comparative Psychology" OR DE "Consulting Psychology" OR DE "Correctional Psychology" OR DE "Cross Cultural Psychology" OR DE "Depth Psychology" OR DE "Developmental Psychology" OR DE "Ecological Psychology" OR DE "Evolutionary Psychology" OR DE "Experimental Psychology" OR DE "Feminist Psychology" OR DE "Folk Psychology" OR DE "Forensic Psychology" OR DE "Geropsychology" OR DE "Health Care Psychology" OR DE "Humanistic Psychology" OR DE "International Psychology" OR DE "Mathematical Psychology" OR DE "Metapsychology" OR DE "Neuroeconomics" OR DE "Physiological Psychology" OR DE "Positive Psychology" OR DE "Psychodynamics" OR DE "Psychology of Men" OR DE "Psychology of Women" OR DE "Self Psychology" | 124433 |
| #4 | DE "Psychiatric Evaluation" OR DE "Forensic Evaluation" OR DE "Cognitive Assessment" OR DE "Achievement Measures" OR DE "Aptitude Measures" OR DE "Comprehension Tests" OR DE "Executive Functioning Measures" OR DE "Intelligence Measures" OR DE "Memory and Learning Measures" OR DE "Mental Health and Illness Assessment" OR DE "Psychodiagnosis" OR DE "Psychodiagnostic Interview" OR DE "Psychodiagnostic Typologies" OR DE "Psychological Autopsy" OR DE "Psychological Report" OR DE "Psychodiagnostic Interview" OR DE "Diagnostic Interview Schedule" OR DE "Structured Clinical Interview" OR DE "Psychological Assessment" OR DE "Attitude Measures" OR DE "Behavioral Assessment" OR DE "Cognitive Assessment" OR DE "Communication and Language Measures" OR DE "Creativity Measurement" OR DE "Developmental Measures" OR DE "Emotional Assessment" OR DE "Health Psychology Assessment" OR DE "Human Computer Interaction Measures" OR DE "Human Factors Measures" OR DE "Implicit Measures" OR DE "Mental Health and Illness Assessment" OR DE "Motivation Measures" OR DE "Neuropsychological Assessment" OR DE "Nonsubstance Related Addiction Measures" OR DE "Perceptual Measures" OR DE "Personality Measures" OR DE "Preference Measures" OR DE "Psychodiagnostic Measures" OR DE "Psychosocial Assessment" OR DE "Sex and Gender Measures" OR DE "Social and Interpersonal Measures" OR DE "Stress and Coping Measures" OR DE "Substance Abuse and Addiction Measures" OR DE "Psychological Report" OR DE "Screening Tests" OR DE "Psychological Screening Inventory" | 113623 |
| #5 | DE "Sexology" | 450 |
| #6 | TI ( “mental health” OR “mental hygiene” OR “mental status” OR “mental care” OR “mental condition” OR psychosocial OR psycholog* OR psychiatr* OR psychotherap* OR neuropsych* OR neurocognitive OR psychometric OR sexolog* ) OR AB ( “mental health” OR “mental hygiene” OR “mental status” OR “mental care” OR “mental condition” OR psychosocial OR psycholog* OR psychiatr* OR psychotherap* OR neuropsych* OR neurocognitive OR psychometric OR sexolog* ) | 1105639 |
| #7 | S1 OR S2 OR S3 OR S4 OR S5 OR S6 | 1213781 |
| #8 | DE "Transgender" | 5,546 |
| #9 | DE "Gender Dysphoria" | 1016 |
| #10 | DE "Transsexualism" | 3,395 |
| #11 | TI ( transgender* OR transsex* OR “trans patient” OR “trans patients” OR “trans person” OR “trans persons” OR “trans people” OR “gender non-conforming” OR “non-binary” OR “nonbinary” OR “gender dysphoria” OR “gender identity disorder” OR “gender identity disorders” OR “genderqueer” OR “gender incongruence” OR “gender incongruences” OR “gender incongruencies” OR “gender incongruity” OR “gender incongruities” OR “male to female” OR “female to male” OR FTM OR MTF OR transmasculine OR transfeminine ) OR AB ( transgender* OR transsex* OR “trans patient” OR “trans patients” OR “trans person” OR “trans persons” OR “trans people” OR “gender non-conforming” OR “non-binary” OR “nonbinary” OR “gender dysphoria” OR “gender identity disorder” OR “gender identity disorders” OR “genderqueer” OR “gender incongruence” OR “gender incongruences” OR “gender incongruencies” OR “gender incongruity” OR “gender incongruities” OR “male to female” OR “female to male” OR FTM OR MTF OR transmasculine OR transfeminine ) | 70,580 |
| #12 | S9 OR S10 OR S11 OR S12 | 71916 |
| #13 | DE "Surgery" OR DE "Amputation" OR DE "Bariatric Surgery" OR DE "Circumcision" OR DE "Cochlear Implants" OR DE "Colostomy" OR DE "Dental Surgery" OR DE "Endocrine Gland Surgery" OR DE "Gender Reassignment" OR DE "Heart Surgery" OR DE "Hysterectomy" OR DE "Induced Abortion" OR DE "Neurosurgery" OR DE "Organ Transplantation" OR DE "Plastic Surgery" OR DE "Postsurgical Complications" OR DE "Stereotaxic Techniques" OR DE "Sterilization (Sex)" OR DE "Transection" | 33798 |
| #14 | TI ( surg* OR operat* OR presurg* OR preoperat* OR Vaginoplast* OR Phalloplast* OR “chest reconstruction” OR “breast reconstruction” OR “chest masculinization” OR Mastectom* OR “breast augmentation” OR Orchiectom* OR orchidectomy* OR clitoroplast* OR labiaplast* OR Hysterectom* OR Oophorectom* OR Penectom* OR scrotoplast* OR urethroplast* OR vaginectom* ) OR AB ( surg* OR operat* OR presurg* OR preoperat* OR Vaginoplast* OR Phalloplast* OR “chest reconstruction” OR “breast reconstruction” OR “chest masculinization” OR Mastectom* OR “breast augmentation” OR Orchiectom* OR orchidectomy* OR clitoroplast* OR labiaplast* OR Hysterectom* OR Oophorectom* OR Penectom* OR scrotoplast* OR urethroplast* OR vaginectom* ) | 195714 |
| #15 | S13 OR S14 | 211172 |
| #16 | S12 AND S15 | 3474 |
| #17 | DE "Gender Reassignment" | 582 |
| #18 | TI ( “gender affirm” OR “gender affirmation” OR “gender affirming” OR “gender reaffirm” OR “gender reaffirmation” OR “gender reaffirming” OR “sex affirm” OR “sex affirmation” OR “sex affirming” OR “gender confirm” OR “gender confirmation” OR “gender confirming” OR “sex confirm” OR “sex confirming” OR “sex confirmation” OR “gender reassignment” OR “sex reassignment” OR “genital reassignment” OR “gender reassignments” OR “sex reassignments” OR “genital reassignments” OR “sex change” OR “sex changes” OR “medical transition” OR “medical transitions” ) OR AB ( “gender affirm” OR “gender affirmation” OR “gender affirming” OR “gender reaffirm” OR “gender reaffirmation” OR “gender reaffirming” OR “sex affirm” OR “sex affirmation” OR “sex affirming” OR “gender confirm” OR “gender confirmation” OR “gender confirming” OR “sex confirm” OR “sex confirming” OR “sex confirmation” OR “gender reassignment” OR “sex reassignment” OR “genital reassignment” OR “gender reassignments” OR “sex reassignments” OR “genital reassignments” OR “sex change” OR “sex changes” OR “medical transition” OR “medical transitions” ) | 1267 |
| #19 | S17 OR S18 | 1492 |
| #20 | S16 OR S19 | 4009 |
| #21 | S7 AND S20 | **1394** |

| **Scopus** | | |
| --- | --- | --- |
| **#** | **Search** | **Result** |
|  |  |  |
| 1 | TITLE-ABS-KEY ( ( "mental health" OR "mental hygiene" OR "mental status" OR "mental care" OR "mental condition" OR psychosocial OR psycholog* OR psychiatr* OR psychotherap* OR neuropsych* OR neurocognitive OR psychometric* OR sexolog* ) AND ( ( ( "transgender*" OR "transsex*" OR "trans patient*" OR "trans person*" OR "trans people" OR "gender non-conforming" OR "non-binary" OR "nonbinary" OR "gender dysphoria" OR "gender identity disorder*" OR "genderqueer" OR "gender incongru*" OR "male to female" OR "female to male" OR ftm OR mtf OR transmasculine OR transfeminine ) AND ( surg* OR operat* OR presurg* OR preoperat* OR vaginoplast* OR phalloplast* OR "chest reconstruction" OR "breast reconstruction" OR "chest masculinization" OR mastectom* OR "breast augmentation" OR orchiectom* OR orchidectomy* OR clitoroplast* OR labiaplast* OR hysterectom* OR oophorectom* OR penectom* OR scrotoplast* OR urethroplast* OR vaginectom* ) ) OR ( "gender affirm*" OR "gender reaffirm*" OR "sex affirm*" OR "gender confirm*" OR "sex confirm*" OR "gender reassignment*" OR "sex reassignment*" OR "genital reassignment*" OR "sex change*" OR "medical transition*" ) ) ) | **2182** |

| **Dissertations and Thesis Global** | | |
| --- | --- | --- |
| **#** | **Search** | **Result** |
| S1 | ti("mental health" OR "mental hygiene" OR "mental status" OR "mental care" OR "mental condition" OR psychosocial OR psycholog* OR psychiatr* OR psychotherap* OR neuropsych* OR neurocognitive OR psychometric* OR sexolog* ) OR ab("mental health" OR "mental hygiene" OR "mental status" OR "mental care" OR "mental condition" OR psychosocial OR psycholog* OR psychiatr* OR psychotherap* OR neuropsych* OR neurocognitive OR psychometric* OR sexolog* ) | 199955 |
| S2 | ti(( "transgender*" OR "transsex*" OR "trans patient*" OR "trans person*" OR "trans people" OR "gender non-conforming" OR "non-binary" OR "nonbinary" OR "gender dysphoria" OR "gender identity disorder*" OR "genderqueer" OR "gender incongru*" OR "male to female" OR "female to male" OR ftm OR mtf OR transmasculine OR transfeminine ) AND ( surg* OR operat* OR presurg* OR preoperat* OR vaginoplast* OR phalloplast* OR "chest reconstruction" OR "breast reconstruction" OR "chest masculinization" OR mastectom* OR "breast augmentation" OR orchiectom* OR orchidectomy* OR clitoroplast* OR labiaplast* OR hysterectom* OR oophorectom* OR penectom* OR scrotoplast* OR urethroplast* OR vaginectom* )) OR ab(( "transgender*" OR "transsex*" OR "trans patient*" OR "trans person*" OR "trans people" OR "gender non-conforming" OR "non-binary" OR "nonbinary" OR "gender dysphoria" OR "gender identity disorder*" OR "genderqueer" OR "gender incongru*" OR "male to female" OR "female to male" OR ftm OR mtf OR transmasculine OR transfeminine ) AND ( surg* OR operat* OR presurg* OR preoperat* OR vaginoplast* OR phalloplast* OR "chest reconstruction" OR "breast reconstruction" OR "chest masculinization" OR mastectom* OR "breast augmentation" OR orchiectom* OR orchidectomy* OR clitoroplast* OR labiaplast* OR hysterectom* OR oophorectom* OR penectom* OR scrotoplast* OR urethroplast* OR vaginectom* )) | 449 |
| S3 | ti("gender affirm*" OR "gender reaffirm*" OR "sex affirm*" OR "gender confirm*" OR "sex confirm*" OR "gender reassignment*" OR "sex reassignment*" OR "genital reassignment*" OR "sex change*" OR "medical transition*" ) OR ab("gender affirm*" OR "gender reaffirm*" OR "sex affirm*" OR "gender confirm*" OR "sex confirm*" OR "gender reassignment*" OR "sex reassignment*" OR "genital reassignment*" OR "sex change*" OR "medical transition*" ) | 266 |
| S4 | S2 OR S3 | 653 |
| S5 | S1 AND S4 | **93** |
